# Fabrication and Application of Photocatalytic Composites and Water Treatment Facility Based on 3D Printing Technology

**DOI:** 10.3390/polym13132196

**Published:** 2021-07-01

**Authors:** Zhirui Mai, Di Liu, Ziyao Chen, Dongsong Lin, Wenxu Zheng, Xianming Dong, Qiongzhi Gao, Wuyi Zhou

**Affiliations:** 1 Biomass 3D Printing Materials Research Center, College of Materials and Energy, South China Agricultural University, Guangzhou 510642, China; maizhirui@stu.scau.edu.cn (Z.M.); 15507483354@163.com (D.L.); FrankChen0108@163.com (Z.C.); LDS17724116470@163.com (D.L.); wzheng@scau.edu.cn (W.Z.); dongxming@263.net (X.D.); 2 Key Laboratory for Biobased Materials and Energy of Ministry of Education, Guangzhou 510642, China; 3 Department of Pharmaceutical Engineering, College of Materials and Energy, South China Agricultural University, Guangzhou 510642, China

**Keywords:** 3D printing, water treatment equipment, polylactic acid, titanium dioxide, composites, photocurable coating

## Abstract

Currently, the degradation of organic pollutants in wastewater by photocatalytic technology has attracted great attention. In this study, a new type of 3D printing material with photocatalytic activity was first prepared to print a water treatment equipment, and then a layer of silver-loaded TiO_2_ was coated on the equipment to further improve the catalytic degradation performance. The composite filaments with a diameter of 1.75 ± 0.05 mm were prepared by a melt blending method, which contained 10 wt% of modified TiO_2_ and 90 wt% of PLA. The silver-loaded TiO_2_ was uniformly coated on the equipment through a UV-curing method. The final results showed that those modified particles were uniformly dispersed in the PLA matrix. The stable printing composite filaments could be produced when 10 wt% TiO_2_ was added to the PLA matrix. Moreover, the photocatalytic degradation performance could be effectively improved after 5 wt% of silver loading was added. This novel facility showed good degradability of organic compounds in wastewater and bactericidal effect, which had potential applications for the drinking water treatment in the future.

## 1. Introduction

In recent years, the situation of water resources is not optimistic [1], as well as the water treatment of sewage and waste water is an urgent problem [2]. Most of the existing water treatment equipment is applied in the industrial production and large-scale wastewater treatment [3,4], but the water treatment equipment for family units or small individual enterprises is not popular enough [5,6]. On the one hand, people’s environmental awareness is relatively weak. On the other hand, the production process of small-scale water treatment equipment is complicated and expensive. Furthermore, the method of industrial-grade wastewater treatment is not suitable for the use of small-scale equipment [7,8,9,10].

**Figure 1:**
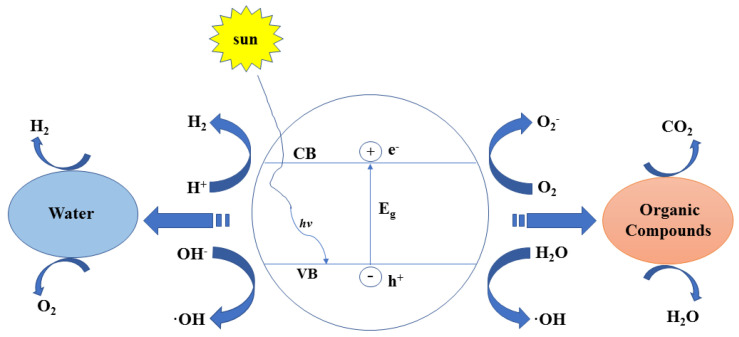
Schematic diagram of the principle of photocatalytic degradation of organic matter.

Photocatalysis is widely used in organic matter degradation and sterilization [11]. In the degradation of organic pollutants, photocatalytic technology can effectively degrade the pollutants into small molecules such as carbon dioxide, water, and nitrogen [12]. Therefore, the secondary pollution to the environment is fundamentally eliminated. TiO_2_ is the most common photocatalyst due to its low cost, good photosensitivity, and resistance to light corrosion [13]. However, its forbidden band width is larger, thus metal doping is often used to reduce its band gap, thereby improving its catalytic performance. Ag, TiO_2_, and graphene were used for Hajipour et al. [14] to make composites that have good photocatalytic degradation activity for organics. In their review, the photocatalytic decomposition of various organic compounds in water is clearly demonstrated by Matthews et al. [15], showing that TiO_2_ photocatalysis can completely oxidatively degrade hydrocarbons, surfactants, and organic pesticides in water. The principle of photocatalytic degradation of organic matter is shown in Figure 1.

At present, semiconductor inorganic materials are mainly used in photocatalytic technology as a catalyst, and light is used as energy to carry out redox reactions to degrade organic matter [16,17]. It is clearly mentioned in the review by Kanmani et al. [18] that the form of loading method or direct input method is usually adopted by photocatalytic reactors. However, the method of directly inputting semiconductor materials is difficult to recycle and cannot be reused. As for the loading process, the manufacturing process is often complicated and cumbersome. In addition, the production cost and cycle are relatively long, which greatly limit the treatment methods of sewage and wastewater.

The 3D printing technology, also known as additive manufacturing, is a technology that uses adhesive materials such as engineering plastics, photosensitive resins, and powdered metals based on digital model files to construct three-dimensional objects through layer-by-layer printing [19]. As an emerging technology, the 3D printing technology has many advantages compared with the traditional technology: The product is printed at one time, the production process is simple, and the production cycle is short. Second, the cost is lower, especially in a small batch production, which has a more significant cost advantage than traditional manufacturing [20,21]. Nowadays, the 3D printing technology is increasingly used in water treatment equipment. In their review, Balogun et al. [22] detailed the application of 3D printing in the manufacture of partitions and membranes for water treatment modules. Martin-Somer et al. [23] developed a high-performance and low-cost solar collector for water treatment manufactured by 3D printing technology. Moreover, Hwa et al. [24] reported a porous ceramic membrane made by 3D printing technology for water filtration. From this point of view, the research and application of 3D printing in the water treatment has attracted more and more attention.

In response to the above problems, this research aims to use 3D printing to produce the water treatment equipment that can efficiently treat wastewater quickly, conveniently, and inexpensively, so as to achieve the purpose of the convenient production of equipment and obvious effects of sewage and wastewater treatment. The self-designed water treatment equipment was adopted in this subject. Starting from the reaction form of the equipment and the special structure constructed, the photocatalytic technology and 3D printing technology were combined to prepare the TiO_2_/PLA composites with the photocatalytic activity and 3D printing function. In addition, the Ag/TiO_2_ photocurable coating with the photocatalytic activity was prepared, which improved the performance of the 3D printing water treatment equipment. On the one hand, it can solve the production and function problems of water treatment equipment at the same time. On the other hand, the use of 3D printing as a manufacturing method can help researchers more conveniently study the impact of the components and structures of equipment on water treatment efficiency. Through the research of this subject, the application direction of 3D printing will be broadened, and the practical value of photocatalysis and 3D printing in society will be promoted. This is of great significance in terms of water pollution and the future development of 3D printing.

## 2. Experimental

### 2.1. Materials Required

Polylactic acid (PLA, grade 4032D), purchased from Nature Works company (Blair, NE, USA), has a density of 1.24 g/cm^3^ and a melt flow index of 7 g/10 min. Lipophilic TiO_2_ (average particle size 100 nm, purity 98.3%) was bought from Guangzhou Riyou Technology Co., Ltd. (Guangzhou, China). The silane coupling agent (KH-570) was purchased from United Carbon Corporation (Danbury, CT, USA). The 1,6-hexanediol diacrylate (HDDA) was bought from Shanghai Shucan Industrial Co., Ltd. (Shanghai, China). The tripropylene glycol diacrylate (TPGDA) was purchased from Hubei Hengjingrui Chemical Co., Ltd. (Yingcheng, China). Polyurethane acrylate (423) was gained from Shenzhen Hebang New Material Technology Co., Ltd. (Shenzhen, China). Phenylbis (2, 4, 6-trimethylbenzoyl) phosphine oxide (819) was acquired from Zibo Paiya Chemical Technology Co., Ltd. (Zibo, China). In addition, 2,4,6-trimethylbenzoyl diphenylphosphine oxide (TPO) was obtained from Hubei Qifei Pharmaceutical Chemical Co., Ltd. (Tianmen, China). Moreover, 2-hydroxy-2-methyl-1-phenyl-1-acetone (1173) was produced from Shanghai Shucan Industrial Co., Ltd. (Shanghai, China). Acryloyl morpholine (ACMO) was gained from Shanghai Shucan Industrial Co., Ltd. (Shanghai, China). None of the above reagents had been purified further. Furthermore, the composition and shape of the main matrix materials have been indicated, and all other materials are pure reagents.

### 2.2. Experimental Methods

#### 2.2.1. Preparation of Nano-TiO_2_ Modified by the Silane Coupling Agent

**Figure 2:**
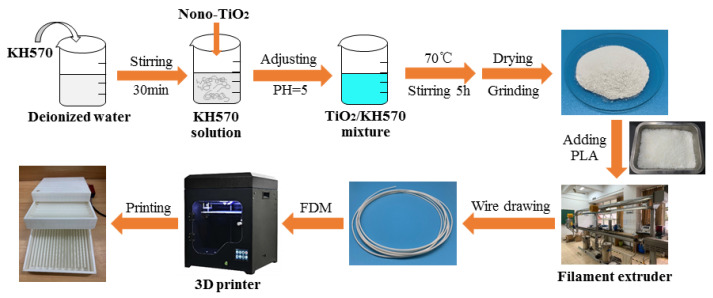
Modified TiO_2_ preparation process, material production, and finished product printing process.

A certain amount of KH570 was added to the deionized water, and stirred magnetically for 30 min. After complete dissolution, the TiO_2_ powder was added to ensure m (KH570): m (TiO_2_) = 0.3. NaOH or HCl was used to adjust the pH of the solution to 5. After stirring for 5 h at 70 °C, the resulting product was centrifuged and then washed with deionized water 3 times. Finally, it was dried and ground in a vacuum drying oven at 80 °C to obtain the modified TiO_2_ powder. The preparation process, material production, and finished product printing process of modified TiO_2_ were shown in Figure 2.

#### 2.2.2. Preparation of TiO_2_(KH-570)/PLA Composites

The required PLA and nano-TiO_2_ powder was placed in an oven and heated at 80 °C for 3 h. Then, 300 g of dry PLA was taken and mixed with a certain proportion (such as 1%, 3%, 5%, and 10%) of TiO2(KH-570) and added to the mixer. The speed of the mixer was set to 100 rpm. The mixture was prepared after starting for 10 min and mixing. Moreover, the mixture material was blended and granulated by a twin-screw extruder (SESI-2025, Beijing Pu Analysis General Instrument Co., Ltd., Beijing, China), and the temperature of each zone of the extruder was set to 175, 176, 177, 176, and 176 °C, respectively. The main engine speed was set to 28 r/min, and the feeding speed was 15 r/min. The resulting blend was dried in an oven at 80 °C for 3 h, and then made into a 3D printing wire (diameter = 1.75 mm) using a single-screw extruder. In addition, the extrusion temperature was 175 °C, and the pulling speed was 60 r/min.

#### 2.2.3. Preparation of Silver-Loaded TiO_2_ Photocurable Coatings

The nano-TiO_2_ powder was placed in deionized water, stirred for 10 min, and ultrasonicated for 3 min until it was completely dispersed. In addition, an appropriate amount of the EDTA solution was added dropwise to adjust the pH of the TiO_2_ aqueous solution to about 6. Afterwards, 1.6 g of AgNO_3_ was dissolved in deionized water to prepare 425 mL of the silver nitrate solution (0.022 mol/L). Under the condition of passing N_2_, the silver nitrate solution was added to the TiO_2_ aqueous solution, and then the mixed solution was continuously stirred under the irradiation of the ultraviolet lamp with 325 nm of wavelength. After about 30 min, the stirring continued until the solution was layered and settled. After removing the supernatant, the precipitate was filtered and washed, and the filter cake was washed with deionized water until the pH was neutral. The filter cake was dried in a dryer at 110 °C, pulverized, and then processed by a ball mill to obtain the TiO_2_-Ag composite photocatalysts. The silver-loaded TiO_2_ composite with different contents was prepared according to different concentrations of sodium nitrate solution where the mass fraction was 1%, 2%, 3%, and 5%, respectively. Twenty grams of reactive diluent HDDA and 50 g of TPGDA were added, respectively, and mixed in a breaker. Then, 30 g of polyurethane acrylate was added and stirred at 40 °C for 20 min until mixed completely. The mass fraction of the silver-loaded TiO_2_ photocatalysts with different concentrations (such as 1%, 2%, and 3%) and a photo-initiator of 3% were added, respectively. The UV curing resins with silver-loaded TiO_2_ photocatalyst was obtained by magnetic stirring for 30 min in the dark. Then, these UV curing resins were coated on the surface of the printed facility substrate by a brush coating method, and then moved to near an ultraviolet radiation chamber for UV curing reaction until completely cured.

#### 2.2.4. The 3D Printing Test

The printing performance of the TiO_2_(KH-570)/PLA 3D printing composites was tested with an FDM 3D printer (FS-200, Guangzhou Feisheng Intelligent Technology Co., Ltd., Guangzhou, Guangdong, China). The model used for 3D printing was designed using Auto CAD 2014 (Autodesk, Sylvania, OH, USA). In addition, a file was exported in stl format and imported to Cura (Version 15.02.1, Ultimaker Corp., Utrecht, Netherlands). Different printer parameters were adjusted using the control variable method. The set parameters were shown in Table 1. At last, the printing effect was evaluated and the best printing parameters were determined.

### 2.3. Characterization

#### 2.3.1. FTIR Analysis

The material was characterized by a Fourier transform infrared spectrometer (360.E.S.P, Nicolet), with the spectral range of 4000–400 cm^−1^. The scanning resolution was set to 4 cm^−1^. Furthermore, the attenuated total reflection mode was used for 32 scans per sample.

#### 2.3.2. SEM Analysis

The scanning electron microscope (XL-30E, Philip Electronic Optics Co., Ltd.; Amsterdam, The Netherlands) was used to observe the surface structure of the sample which was printed with composites. The samples were quenched by liquid nitrogen, and their surface structure were observed. Before the test, the cross-section of the gold sprayed sample with a thickness of 12 nm was used as the conductive layer. The acceleration current of the sprayed gold was 30 mA, the gold content used was 99.99%, as well as the sputtering distance was 6 cm.

#### 2.3.3. TG Analysis

The thermogravimetric analyzer (DTG-60, Shimadzu Co., Ltd., Kyoto, Japan) was applied to test the material properties from 25 to 500 °C at a rate of 10 °C under argon protection, in which the argon flow rate was 20 mL/min.

#### 2.3.4. X-ray Analysis

An X-ray diffractometer (D8 ADVANCE, Bruker AXS; Karlsruhe, Germany) was used to evaluate the crystal phase of the sample. The measurement was carried out in the 2θ range of 5–90°, with a scan rate of 5°/min, using copper target radiation (λ = 1.542) operating at 30 kV and 20 mA.

#### 2.3.5. Degradation Performance Test

The methylene blue solution was used to simulate organic pollutants. In this experiment, the water flow rate was adjusted to 5~10 L/h, and the initial concentration of methylene blue solution was 10 mg/L. Under the irradiation of a 30 W ultraviolet lamp, samples were taken every 30 min and analyzed by an ultraviolet-visible spectrophotometer (UV-2520, Shimadzu Co., Ltd., Kyoto, Japan) at the absorption wavelength of 662 nm. The formula for calculating the degradation rate (*η*) of methylene blue solution is expressed as follows:

*η* = (*A*_0_ − *A_t_*)/*A*_0_ × 100% (1)

where *A*_0_ is the initial absorbance and *A_t_* is the absorbance after the degradation reaction.

#### 2.3.6. Sterilization Performance Test

The experiment was set up of four control groups as follows: The first was the water treatment equipment for raw water without the catalyst and UV lamp. The second was the polylactic acid printing water treatment equipment that turns on the UV lamp. The third was the TiO_2_(KH-570)/PLA material printing water treatment equipment under UV light. The last one was the TiO_2_(KH-570)/PLA material printing water treatment equipment coated with silver-loaded TiO_2_ light-cured coating under the UV lamp.

The liquid culture medium was prepared as follows: 10 g peptone, 10 g NaCl, and 5 g yeast powder were weighed, the materials were mixed and stirred, diluted with 1 L deionized water, and then placed in an ultrasonic water bath for 3 min. After being completely dissolved, NaOH or HCl was used to adjust the pH of the solution to around 7. The mixed solution was separately added into two 250 mL conical flasks to ensure that the bottle body was completely wrapped and sealed with kraft paper [25,26]. The wrapped conical flask was placed in an autoclave for sterilization at 121 °C for 30 min. After the sterilization was completed, it was placed at room temperature for 5 h before use.

The *E. coli* strains were taken out of the laboratory low-temperature storage box and moved to a 4 °C environment to thaw for 2 h. The thawed strain was measured and added to the previously prepared liquid culture medium under aseptic conditions by measuring 1 mL, and again sealed, banged, and oscillated in a constant temperature shaker at 37 °C at a speed of 120 r/min for 12 h. At this time, the *E. coli* hydroponic culture was completed. Subsequently, the cultured solution was placed in a centrifuge tube at 8000 rpm and centrifuged for 5 min, and sterilized 0.9% NaCl saline was added to be washed and centrifuged 3 times to obtain a precipitated gray powder film, which was stored in a refrigerator at −8 °C [27].

Sterilization experiments were carried out on the four control samples, respectively. The plate counting method described in ISO 20143 was used to determine the photocatalytic antibacterial effect of different samples [28]. In simple terms, the initial number of bacteria present after incubation was calculated by counting the number of colonies in a 10-fold dilution.

## 3. Results and Discussion

### 3.1. FTIR Analysis

**Figure 3:**
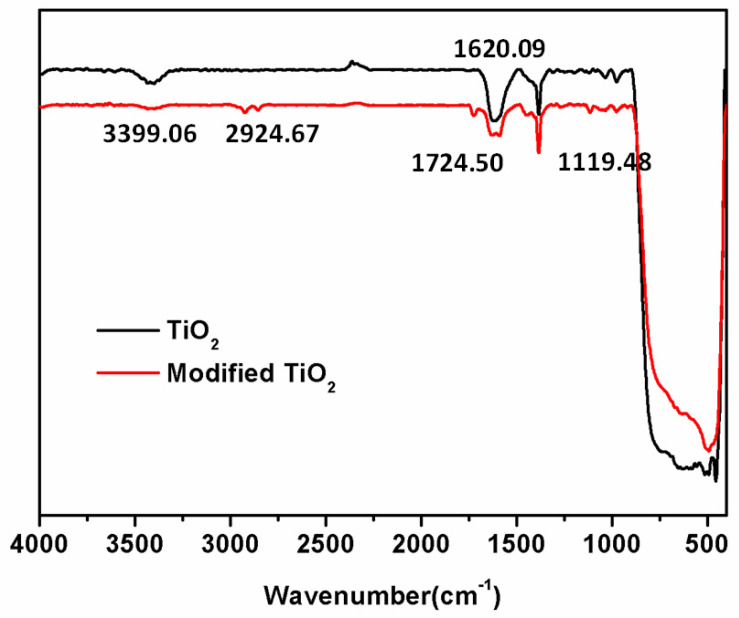
Infra-red spectrum of TiO_2_ before and after modification.

Nano-TiO_2_ was modified with the silane coupling agent KH-570, as well as the samples before and after modification were analyzed by infrared spectrometer, as shown in Figure 3. Among them, the wide absorption band of 3339.06 cm^−1^ is the -OH absorption band on the surface of TiO_2_, and the 500–800 cm^−1^ is the vibration absorption peak of the framework of Ti-O-Ti, this is in line with the reports in the literature [29]. Relative to the unmodified TiO_2_, the absorption peak at 2924.67 cm^−1^ on the infrared spectrum of TiO_2_ modified by the silane coupling agent KH-570 is the stretching vibration peak of –CH_3_ and –CH_2_ [30]. According to reports from Li et al. [31], the characteristic peaks of stretching vibration appearing at 1724.50 and 1620.09 cm^−1^ are the characteristic peaks of the C=O and C=C groups in the silane coupling agent. After being modified by the silane coupling agent, a new absorption peak appeared at 1119.48 cm^−1^, which is the stretching vibration absorption peak of Si–O–Si, indicating that the silane coupling agent has been successfully grafted onto TiO_2_ [32].

**Figure 4:**
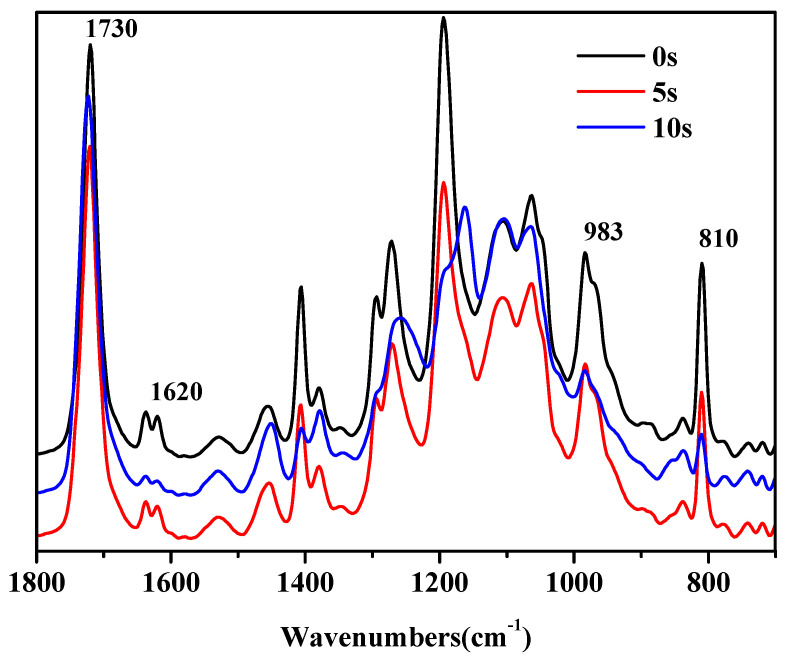
Infra-red spectrum of photosensitive resin under different irradiation times.

An infrared spectrometer was used to perform the real-time infrared analysis on the photosensitive resin of the sample, and continuously monitor the infrared spectrum of the sample under ultraviolet light irradiation, as shown in Figure 4. It is found that the characteristic absorption peak of C=O at the wavenumber of 1730 cm^−1^ is almost consistent with the results by Deng et al. [33]. With the prolonged exposure time of ultraviolet light, the C=C stretching vibration and bending vibration peaks at the wave numbers of 1620 and 810 cm^−1^ gradually weakened, and the bending vibration absorption band outside the 983 cm^−1^ =CH plane also weakened, indicating that the light-curing double bond was gradually opened, and the light-curing reaction was basically completed.

### 3.2. SEM Analysis

**Figure 5:**
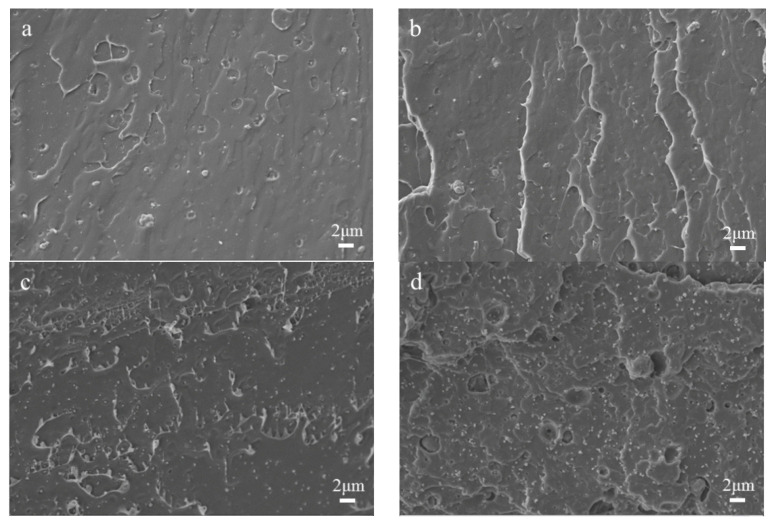
SEM image of TiO_2_ on the PLA cross section before and after modification: (**a**) 1% TiO_2_/PLA; (**b**) 3% TiO_2_/PLA; (**c**) 5% TiO_2_(KH-570)/PLA; (**d**) 10% TiO_2_(KH-570)/PLA.

**Figure 6:**
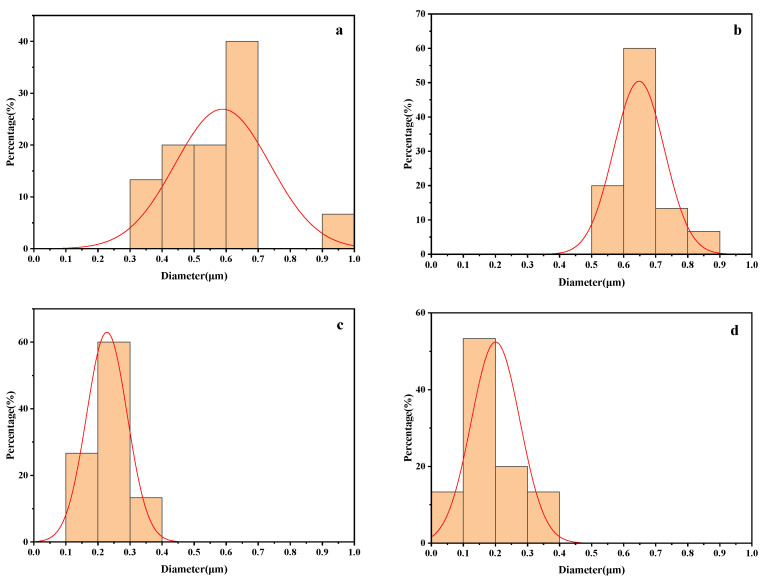
Diameter distribution of TiO_2_ filler: (**a**) 1% TiO_2_/PLA; (**b**) 3% TiO_2_/PLA; (**c**) 5% TiO_2_(KH-570)/PLA; (**d**) 10% TiO_2_(KH-570)/PLA.

The cross-sectional topography of the unmodified TiO_2_/PLA composites at 6000 times magnification is shown in Figure 5a,b. It can be seen that the unmodified TiO_2_ is prone to agglomeration in the PLA, resulting in larger particle aggregates and poor dispersion. This is due to the fact that the unmodified TiO_2_ failed to form a chemical bond with PLA, and there is no interaction [34]. The morphology of the quenched section of the TiO_2_/PLA composites modified by the silane coupling agent KH-570 at a magnification of 6000 times is shown in Figure 5c,d. It is found that the dispersion of the modified TiO_2_ in the PLA is extremely high which is a great improvement, and the distribution in the matrix is more uniform and the size is smaller. This is due to the fact that after the material is coated with the silane coupling agent KH-570, a good steric hindrance is formed between the particles, which reduces the self-cohesive force of TiO_2_. In the figure, the phenomenon of “drawing” appears in Figure 5c, and the surface of the PLA substrate is flat, which may be due to the formation of a hydrogen bond such as the chemical bond between the nano-TiO_2_ modified by the silane coupling agent and the PLA, which increases the binding capacity between the TiO_2_ nanoparticles and the PLA polymer material. The result is generally consistent with the report by Luo et al. [35]. However, there are some cavities and layered structures on the PLA substrate surface in Figure 5d, which indicates that there is a difference in bonding between the modified TiO_2_ substrate and the PLA substrate, especially when the force is uneven during quenching, the TiO_2_ powder and the PLA are separated. Therefore, it can be inferred that with the increase of TiO_2_ content, the stress concentration points in the system increase, which will have a certain impact on the mechanical properties of PLA materials. In order to clearly demonstrate the effect of KH-570 modification on the aggregation and dispersion of TiO_2_, Figure 6 provides the diameter distribution of the TiO_2_ filler before and after the modification. The average diameters of 1% TiO_2_/PLA, 3% TiO_2_/PLA, 5% TiO_2_(KH-570)/PLA, and 10% TiO_2_(KH-570)/PLA filler are 0.59, 0.65, 0.23, and 0.20 μm, respectively. It can be seen that the diameter after modification is significantly smaller than that before modification for the TiO_2_ filler. The result indicates that the modified TiO_2_ nanoparticles are better dispersed in the PLA matrix and not easy to form agglomerations.

**Figure 7:**
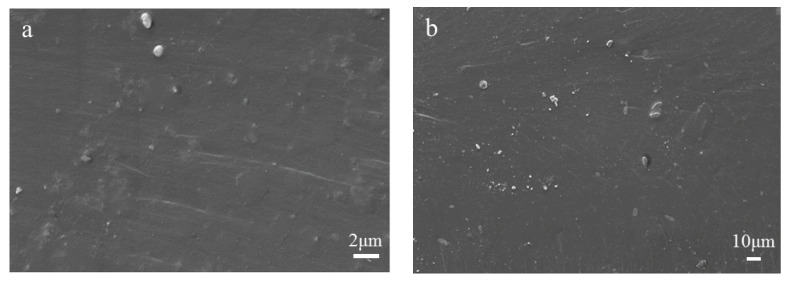

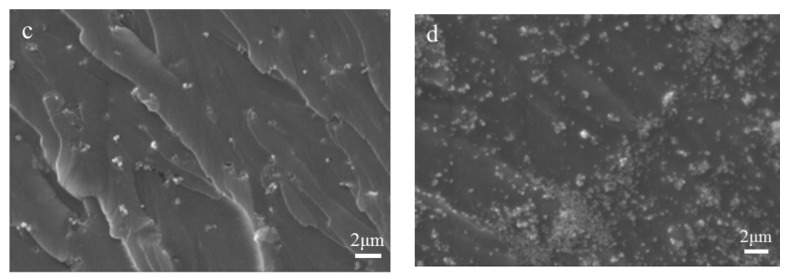
SEM images of quenched sections of silver-loaded TiO_2_ light-cured coatings with different additives: (**a**) 1% Ag-TiO_2_; (**b**) 2% Ag-TiO_2_; (**c**) 3% Ag-TiO_2_; (**d**) 5% Ag-TiO_2_.

The SEM scanning electron microscope image of the cross-section of the spline with different contents of silver-loaded TiO_2_ added to the coating material is shown in Figure 7. Among them, Figure 7a–d are respectively 1%, 2%, 3%, and 5% of the light-curable coating composites loaded with TiO_2_-Ag. By observing the quenched sections of Figure 7a,b, it can be seen that when a small amount of silver-loaded TiO_2_ is added, the surface morphology of the cross section of the system is smooth, indicating that a small amount of inorganic nanoparticles has little effect on the internal structure of the composites. When the content of nanoparticles in the system increases, a small amount of nano-TiO_2_ would agglomerate [36]. The irregular lamellar structure appears on the interrupted surface of Figure 7c, and the edge of the lamellar is jagged, as well as the phenomenon of stress whitening appears, indicating that the system yields when it is fractured, which is a ductile fracture. This phenomenon may be caused by the addition of nano-TiO_2_ that has enhanced the toughness of the material to a certain extent. In addition, Figure 7d is different from Figure 7c, as well as there is no stress whitening phenomenon in Figure 7c. This is due to the fact that with the increase of additives, the material inside is loose.

### 3.3. TG Analysis

**Figure 8:**
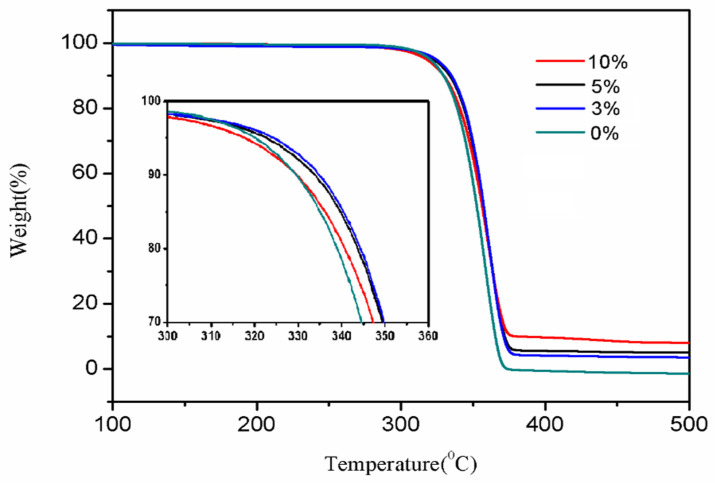
The effect of TiO_2_ content on thermal stability.

The thermal stability performance can be studied by conducting thermal weight loss experiments on PLA and composites with different TiO_2_ content gradient additions. The TiO_2_ content gradient addition and decomposition temperature are shown in Table 2. The heat loss curve of PLA and its composites is shown in Figure 8. From the figure, it can be seen that when adding different contents of TiO_2_, the thermal degradation initial temperature *T*_0_ of the composites is 0.8, 4.2, and 5.3 °C higher than that of the pure PLA, respectively, which is basically in line with the results by Atanasoulia et al. [37]. The end temperature *T_f_* of thermal degradation also increased (about 3.1 °C), indicating that adding a certain content of TiO_2_ can improve the heat resistance of the material. This is due to the addition of KH570 modified TiO_2_ inorganic particles, KH570 molecules, and PLA form entanglements, forming hydrogen bonds. In addition, the formation of Ti-O-Ti bonds between TiO_2_ and PLA can also enhance its thermal stability. However, with the increase of TiO_2_ addition, the increase in *T*_0_ is not obvious, and the addition of 5% and 10% only increases by 1.1 °C. The reason is that when the content of TiO_2_ exceeds a certain proportion, its dispersion in the PLA system will decrease, as well as the compatibility with the material also decreases, which will lead to the deterioration of the thermal stability of the material.

### 3.4. X-ray Analysis

**Figure 9:**
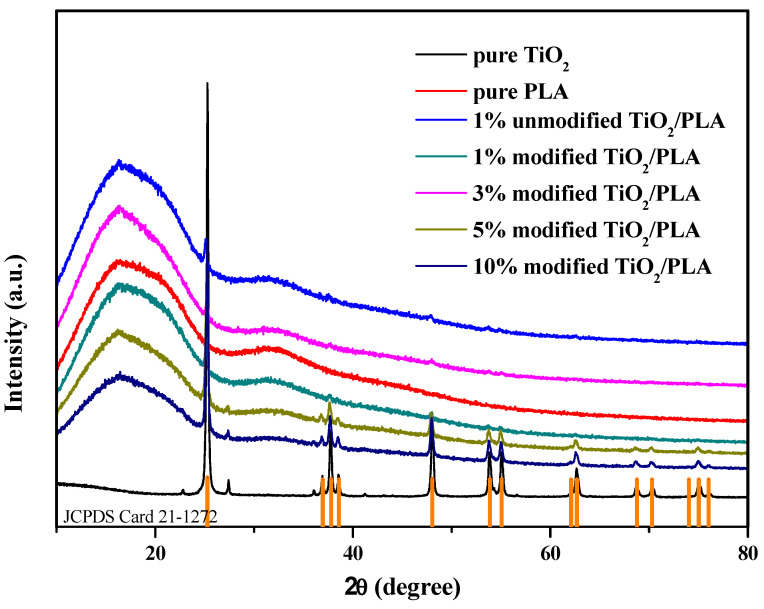
X-ray diffraction pattern of composites.

In Figure 9, the results of the XRD analysis of pure TiO_2_, pure PLA, 1 wt% unmodified TiO_2_/PLA composites, and modified TiO_2_/PLA composites with different concentrations are shown. The phase analysis showed that all the TiO_2_ peaks (JCPDS Card 21-1272) were present in the composites of unmodified TiO_2_ and modified TiO_2_. It is more obvious in samples with a higher TiO_2_ content. As shown in Figure 9, strong diffraction peaks were observed at 25.3, 37.8, 48.0, 53.9, 55.1, and 62.7°. This matches the previous measurement of TiO_2_ [38]. However, in the 1% and 3% composites, the diffraction peaks other than 25.3° are not obvious, which may be caused by the broad peaks of PLA. The above analysis shows that there are TiO_2_ components in the obtained sample.

### 3.5. Printing Test and Degradation Performance Analysis of Water Treatment Equipment

**Figure 10:**
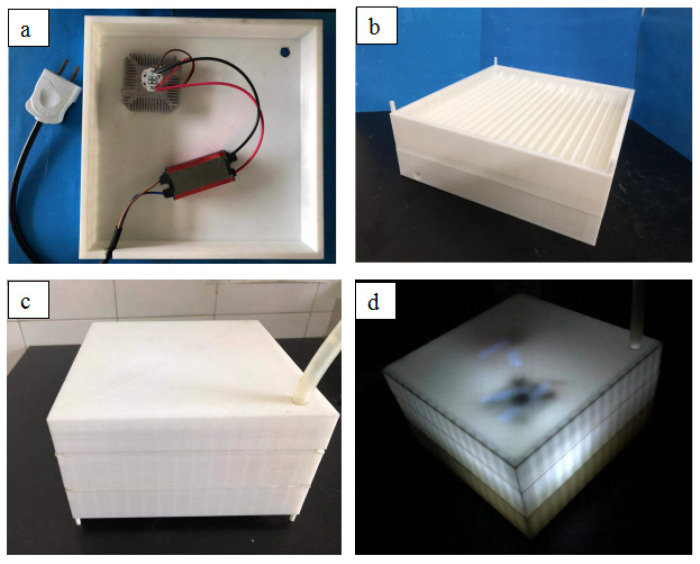
Water treatment equipment diagram: (**a**) UV light source of water treatment equipment; (**b**) photocatalytic unit; (**c**) overall picture of water treatment equipment; (**d**) water treatment equipment operation diagram.

The 10% modified TiO_2_ with the best printing effect was selected, and the TiO_2_/PLA 3D printing composites were prepared. The 3D printing test is carried out according to the parameter range listed in Table 1. Finally, the optimal printing parameters of this material were determined as follows: The nozzle temperature was 205 °C, the printing speed was 40 mm/s, the single-layer printing thickness was 0.2 mm, and the bed temperature was 55 °C. The printing units were assembled into the water treatment equipment. Figure 10 is a schematic diagram of the water treatment equipment.

**Figure 11:**
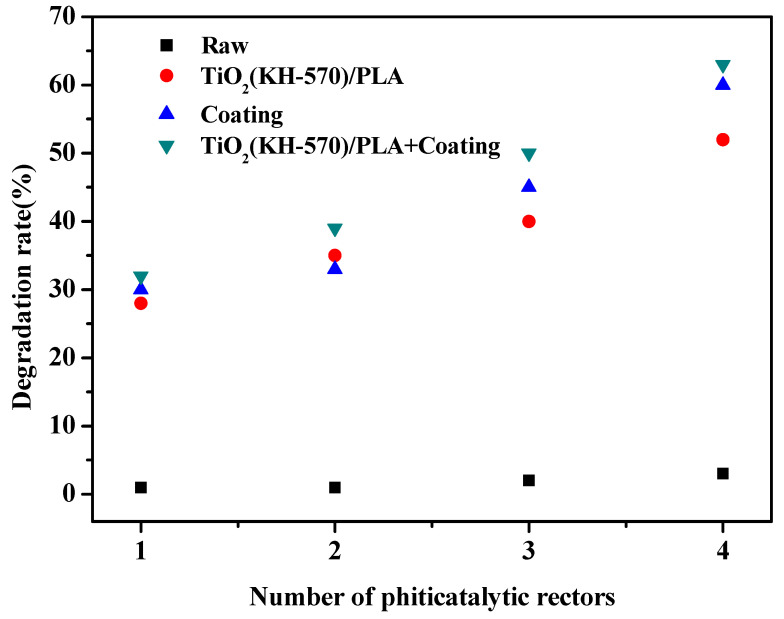
Treatment effect of water treatment equipment on the methylene blue solution.

**Figure 12:**
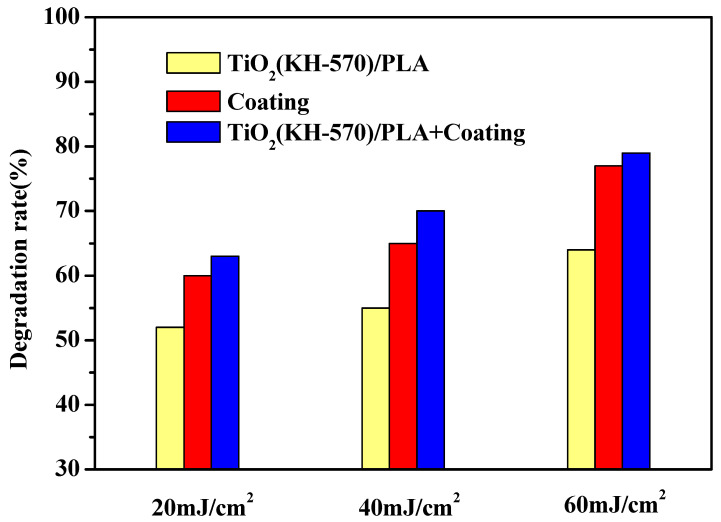
Photocatalytic degradation effects of different light radiation intensities on the methylene blue solution.

The methylene blue solution was used as the simulation target pollutant to carry out photocatalytic degradation efficiency experiments on different composite photocatalytic water treatment equipment. The effects of materials, light intensity, and the number of photocatalytic units on the degradation efficiency of equipment were explored. As shown in Figure 11, the photocatalytic degradation efficiency of multiple catalytic units connected in series with the different materials were tested under the condition that the light intensity was kept at 20 mJ/cm^2^. With the increase in the number of catalytic units, the catalytic degradation efficiency of methylene blue solution by different materials has increased. This is due to the fact that as the number of photocatalytic units increases, the cycle time of pollutants in the reactor increases, which is conducive to a longer contact time between the pollutants and photocatalyst [39,40]. From the experimental data, it can be seen that the water treatment effect after applying the silver-bearing TiO_2_ photocurable coating on the water treatment equipment printed by TiO_2_(KH-570)/PLA composites is improved. On the one hand, since the TiO_2_ in the coating passes the silver loading after the treatment, it will inhibit the photogenerated carrier recombination in the photocatalytic reaction. On the other hand, the more TiO_2_ is distributed per unit area in the coating material, the better the photocatalytic activity, since there are more TiO_2_ nanoparticles distributed in the reactant. It can also be seen that the water treatment effect of the water treatment equipment printed by TiO_2_(KH-570)/PLA composites and coated with TiO_2_ coating composites is better than the other materials. In the case of four catalytic units connected in series, the degradation efficiency of the next cycle reached 63%. Figure 12 shows the degradation effect of methylene blue solution by the water treatment equipment with different substrates under different light radiation intensities and under the condition of four catalytic units connected in series. It is found that the photocatalytic degradation effect increases with the increase of light radiation intensity. The photocatalytic degradation effect reached 79% at 60 mJ/cm^2^.

### 3.6. Printing Test and Degradation Performance Analysis of Water Treatment Equipment

**Figure 13:**
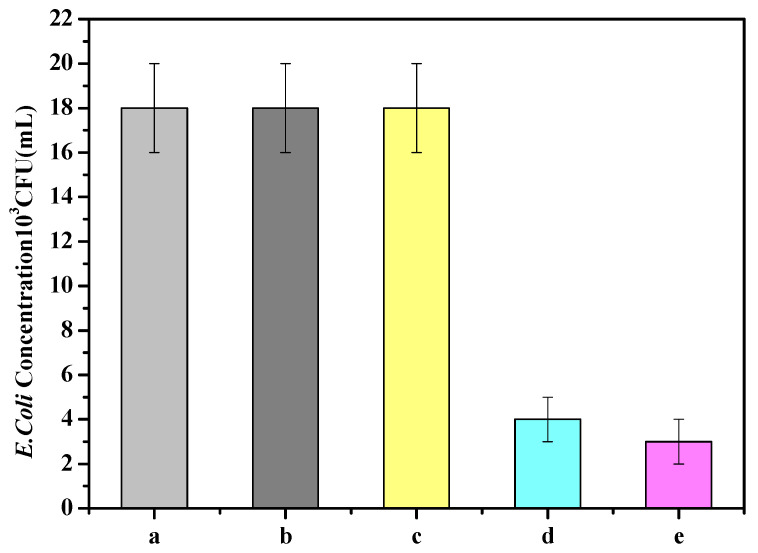
The killing effect of different materials of water processor on *E. coli.* (a) Without the addition of TiO_2_; (b) dark conditions; (c) dark conditions after coating; (d) TiO_2_(KH-570)/PLA printing equipment without coating; (e) TiO_2_(KH-570)/PLA printing equipment with coating.

The results of the experimental comparison of wastewater containing *E. coli* pollutants under the same light intensity are shown in Figure 13. It is found that in the absence of ultraviolet radiation, the concentration of *E. coli* remains basically unchanged after the wastewater flows out, regardless of whether there is titanium dioxide or not. However, in the case of ultraviolet light, the wastewater passes through the containing photocatalyst. The concentration of *E. coli* after the water treatment reactor was lower than that of the water treatment without the photocatalyst, indicating that the presence of TiO_2_ photocatalyst does have a positive effect on the killing of *E. coli*. This is due to the fact that the TiO_2_ in the water treatment device material will generate electron holes and release hydroxyl radicals to enter the bacteria and penetrate the cell wall under the irradiation of the ultraviolet lamp, preventing the transmission of membrane-forming substances, thereby cutting off its respiratory system and electron transport system, and then achieve the effect of sterilization. This sterilization mechanism has been reported by Fonseca et al. [41], and the sterilization effect of our composites is better than theirs. It can also be seen in Figure 13 that the bactericidal effect of TiO_2_/PLA composites is weaker than that of the coating on this basis, since the silver particles on the surface of the coating can be tightly combined with the sulfhydryl group of the enzyme protein in the bacteria to make the protein coagulate, destroying the activity of cell synthase, and causing the cell to lose the ability to divide and die [42,43]. Another important reason is that the TiO_2_ content of the coating is more than that of the composites under the same surface area.

**Figure 14:**
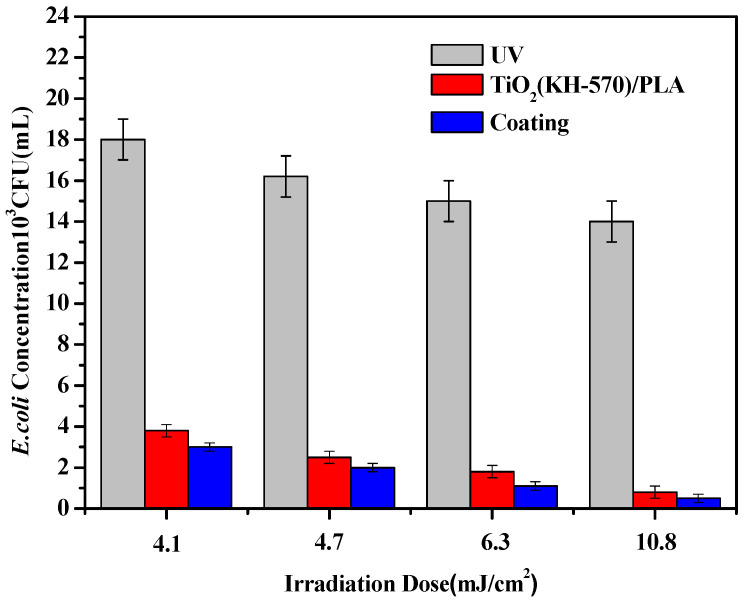
The sterilization effect of different materials of the water processor under different light radiation intensities.

After studying the sterilization effect of water treatment equipment of different materials with the same light intensity on *E. coli*, this experiment explored the effect of light intensity on the killing of *E. coli* by changing different light intensities. As shown in Figure 14, as the light radiation dose gradually increases from 4.1 to 10.8 mJ/cm^2^, it can be seen whether it is under pure ultraviolet light irradiation, TiO_2_/PLA composites or coating treatment. The water treatment reactors all showed a decrease in the concentration of *E. coli* at the outlet. This is due to the fact that as the intensity of light irradiation increases, the energy carried by the ultraviolet light waves is greater. Moreover, it is easier to destroy the nucleic acids in bacteria and viruses under high energy, and to enhance the sterilization effect. In summary, it is also possible to adjust the light intensity to improve the sterilization ability.

## 4. Conclusions

In this work, the dispersion of nano-TiO_2_ was enhanced by the silane coupling agent, and the maximum content reached 10% in the PLA and resin system. The photocatalytic degradation capability of the water processor could be improved by the increased TiO_2_, enhanced ultraviolet light, and low speed water flow. After applying the photocatalytic coating, the water processor owned better photocatalytic degradation efficiency and sterilization activity, and the decomposition of PLA caused by ultraviolet light was prevented. Moreover, the water processor designed in this article was practical and convenient.

## Figures and Tables

**Table 1:** Set 3D printer parameters.

Parameter	Set Value
Filament diameter	1.75 mm
Nozzle temperature	180–220 °C
Nozzle diameter	0.4
Fill density	100%
Printing speed	25–50 mm/s
Single-layer printing thickness	0.1–0.3 mm
Bed temperature	40–70 °C

**Table 2:** TiO_2_ content gradient addition amount.

Sample	*T*_0_/°C	*T_f_*/°C	Δ*T*_0_/°C	Δ*T_f_*/°C
PLA	337.0	368.7		
3% TiO_2_(KH-570)/PLA	337.8	371.8	0.8	3.1
5% TiO_2_(KH-570)/PLA	341.2	371.9	4.2	3.2
10% TiO_2_(KH-570)/PLA	342.3	371.8	5.3	3.1

## Data Availability

Data sharing not applicable.
